# Bee pollens originating from different species have unique effects on ovarian cell functions

**DOI:** 10.1080/13880209.2020.1839514

**Published:** 2020-11-05

**Authors:** Alexander V. Sirotkin, Adam Tarko, Richard Alexa, Alla Fakova, Saleh Alwasel, Abdel Halim Harrath

**Affiliations:** a Department of Zoology and Anthropology, Constantine the Philosopher University in Nitra, Nitra, Slovakia; b Research Institute of Animal Production Nitra, Lužianky, Slovakia; c Department of Zoology, College of Science, King Saud University, Saudi Arabia; d Higher Institute of Applied Biological Sciences of Tunis, University of Tunis El Manar, Tunis, Tunisia

**Keywords:** Viability, proliferation, apoptosis, IGF-I, cell turnover

## Abstract

**Context:**

The species-specific differences and mechanisms of action of bee pollen on reproduction have not been well studied.

**Objective:**

We compared the effects of bee pollen extracts from different plants on ovarian cell functions.

**Materials and methods:**

We compared the effects of pollens from black alder, dandelion, maize, rapeseed, and willow at 0, 0.01, 0.1, 1, 10, or 100 µg/mL on cultured porcine ovarian granulosa cells. Cell viability was assessed with a Trypan blue test, the cell proliferation marker (PCNA), and an apoptosis marker (BAX) were assessed by immunocytochemistry. Insulin-like growth factor (IGF-I) release was measured by an enzyme-linked immunosorbent assay.

**Results:**

Addition of any bee pollen reduced cell viability, promoted accumulation of both proliferation and apoptosis markers, and promoted IGF-I release. The ability of various pollens to suppress cell viability ranked as follows: rapeseed > dandelion > alder > maize > willow. The biological activity of bee pollens regarding their stimulatory action on ovarian cell proliferation ranked as follows: dandelion > willow > maize > alder > rapeseed. Cell apoptosis was promoted by pollens as follows: range > dandelion > alder > rapeseed > willow > maize. The ability of the pollens to stimulate IGF-I output are as follows: willow > dandelion > rapeseed > maize > alder.

**Discussion:**

Bee pollen can promote ovarian cell proliferation by promoting IGF-I release, but it induces the dominance of apoptosis over proliferation and the reduction in ovarian cell viability in a species-specific manner.

**Conclusions:**

This is the first demonstration of adverse effects of bee pollen on ovarian cell viability and of its direct stimulatory influence on proliferation, apoptosis, and IGF-I release. The biological potency of bee pollen is dependent on the plant species.

## Introduction

Bee pollen is flower pollen gathered by the honeybee (*Apis mellifera* L., Apidae) that is then enriched with honey, nectar, and bee salivary enzymes. It contains carbohydrates, proteins, and fatty acids, along with over 250 regulatory molecules including vitamins, minerals, carotenoids, polyphenols (phenolic acids and flavonoids such as quercetin, daidzein, and leukotrienes), phytosterols, and nucleic acids (Denisow and Denisow-Pietrzyk 2016; Cornara et al. 2017; Kieliszek et al. 2018). Due to its putative antimicrobial, antifungal, antioxidant, anti-inflammatory, anti-atherosclerotic, antiaging, antidepressant, anticancer, antidiabetic, hepatoprotective, and hypoglycaemic properties, bee pollen is a promising functional food (Kieliszek et al. 2018) and apitherapeutic drug (Denisow and Denisow-Pietrzyk 2016; Cornara et al. 2017). The large-scale application of bee pollens is complicated by the insufficient knowledge on their physiological effects. For example, available information regarding bee pollen’s effects on reproduction is limited to two studies with contradicting results. Feeding rapeseed bee pollen to rats promoted accumulation of pro-apoptotic (BAX, caspase-3) and anti-apoptotic (BCL-2) proteins in their ovaries, inhibited ovarian production of insulin-like growth factor I (IGF-I), and promoted ovarian release of steroid hormones (Kolesarova et al. 2013). However, in another study, *in vitro* experiments failed to detect any direct impact of rapeseed pollen on proliferation (i.e., accumulation of proliferating cell nuclear antigen [PCNA]), apoptosis (i.e., accumulation of caspase-3), and progesterone release of cultured porcine ovarian granulosa cells, although it did inhibit IGF-I release (Kolesarova et al. 2011a). Therefore, whether bee pollen directly affects ovarian cell proliferation and apoptosis remains unclear. The ratio of proliferation to apoptosis defines ovarian cell viability, which in turn determines ovarian follicle fate (either atresia or ovulation) and the resulting fecundity (Craig et al. 2007; Palma et al. 2012; Monniaux et al. 2016). The action of bee pollens on ovarian cell viability has not yet been explicitly studied. There are also only a few reports (Kolesarova et al. 2011a, 2013) on the influence of bee pollen on IGF-I, which is the key hormonal promoter of cell proliferation, viability, and ovarian hormone release, the main suppressor of apoptosis, and a mediator of the effects of numerous external factors on both non-ovarian and ovarian cells (Quirk et al. 2004; Sirotkin 2011, 2014; Shimizu 2016).

Furthermore, the main difficulty in the application of bee pollen in modern phytomedicine is related to the wide species-specific variation in its composition (Denisow and Denisow-Pietrzyk 2016). Some chemical compounds from pollen of different origins can vary in their content by 10-fold (Donkersley et al. 2017; Kieliszek et al. 2018), and this variation in chemical composition should ultimately affect the biological action of bee pollen. Various bee pollens have been compared in the past, but these were mainly studied in terms of their nutritional value rather than their biological effects (Denisow and Denisow-Pietrzyk 2016; Donkersley et al. 2017; Kieliszek et al. 2018). Comparison of biological activities among various bee pollens in one study would, therefore, enable selection of pollens with maximal apitherapeutic potential. However, we found only one study (Pinto et al. 2010) that compared the antiestrogenic and antigenotoxic activity of two plant species’ bee pollen on yeast and human lymphocytes. No comparison of the effects of bee pollen originating from different plants on ovarian functions has been performed.

This study addresses the following questions: (1) Does bee pollen directly affect ovarian cell viability? (2) Is the effect of pollen on cell viability due to its influence on ovarian cell proliferation, apoptosis, or both? (3) Is the influence of bee pollen on viability, proliferation, and apoptosis due to its effect on IGF-I release? (4) Does the plant species from which bee pollen originates impact its effects on ovarian cells? To address these questions, we compared the dose-dependent effects of bee pollens extracted from five higher plants on the viability, proliferation, apoptosis, and IGF-I production of cultured porcine ovarian granulosa cells. The actions of pollens extracted from black alder (*Alnus glutinosa* L., Betulaceae), dandelion (*Taraxacum officinale* L., Asteraceae), maize (*Zea mays* L., Poaceae), rapeseed (*Brassica napus* L., Brassicaceae), and willow *(Salix* spp. L., Salicaceae), administered at concentrations of 0, 0.01, 0.1, 1, 10, or 100 µg/mL of culture medium, were compared in this study.

## Materials and methods

### Collection and characterisation of bee pollens

Pollen were collected during the beekeeping season (March to August 2016) from the most common plants (i.e., black alder, maize, dandelion, rapeseed, and willow) used by bees in the area around the Institute of Apiculture, Research Institute of Animal Production, Liptovský Hrádok, Slovakia. Pollen loads were collected using pollen traps in beehives. Classification of bee pollen samples by colour (Barth et al. 2010) was performed by comparing the collected pollen with the assembled pollen colour chart (Pollen Identification Color Guide 2016), and the classification was subsequently validated by examining the pollen under a light microscope (Smart et al. 2017). Only samples containing at least 80% pollen of the same species were used in subsequent *in vitro* experiments.

### Isolation and culture of ovarian granulosa cells

Granulosa cells were isolated from the 2 to 5 mm follicles of ovaries of noncycling pubertal Slovakian white gilts that were approximately 180 d of age and originated from the region of Nitra, Slovakia. The cells were then processed and cultured as previously described (Kolesarova et al. 2011a, 2011b; Sirotkin et al. 2012, 2017). Briefly, the cells were cultured in sterile DMEM/F12 1:1 medium (BioWhittaker™, Verviers, Belgium) supplemented with 10% foetal calf serum (BioWhittaker™) and 1% antibiotic-antimycotic solution (Sigma, St. Louis, MO, USA). Cells were plated at a final concentration of 1 × 10^6^ cells/mL of medium in 16-well chamber slides (Nunc Inc., International, Naperville, IL, USA; 200 μL/well) at 37.5 °C in 5% CO_2._ After the formation of a 60–75% confluent monolayer, the medium was replaced (with the same composition as above), and the cells were cultured in the medium supplemented with the extracts of the five different bee pollens listed above at concentrations of 0, 0.01, 0.1, 1, 10, or 100 µg/mL. Bee pollens were first dissolved in 50 µL of dimethyl sulfoxide (DMSO) to make stock solutions of 100 µg pollen/µL, and then, these stock solutions were dissolved in culture media immediately before their addition to the cells, such that the final concentration of DMSO did not exceed 0.01%. Previous studies found no substantial effects of 0.01% DMSO on cell function and viability (Chen and Thibeault 2013; Hebling et al. 2015). Controls included ovarian cells cultured in the incubation medium (with 0.01% DMSO) without bee pollen and medium incubated in the absence of cells (blank control). After two days of culture, cell viability was assessed using a Trypan blue assay. Afterwards, the cells were prepared for immunocytochemical analysis, and the media were stored for use in a subsequent enzyme-linked immunosorbent assay (ELISA), as described previously (Kolesarova et al. 2011a, 2011b; Sirotkin et al. 2012, 2017).

### Immunocytochemical analysis

The PCNA promotes the S-phase of the cell cycle and is expressed during this phase; as such, it serves as a marker of cell proliferation (Wang 2014). BAX (bcl-2-associated X protein) is known to promote apoptosis, so it is used as a cytoplasmic marker of apoptosis (Peña-Blanco and García-Sáez 2018). The presence of these cellular markers of proliferation and apoptosis was detected by immunocytochemistry, as previously described (Kolesarova et al. 2011a, 2011b; Sirotkin et al. 2012, 2017). Specifically, monoclonal primary antibodies against these molecules (Santa Cruz Biotechnology, Inc., Santa Cruz, CA, USA; diluted 1:500 in PBS) and secondary swine antibodies against mouse IgG labelled with horseradish peroxidase (Servac, Prague, Czech Republic; diluted 1:1000) were used. Following incubation with the antibodies, cells were stained with diaminobenzidine (DAB) substrate (Roche Diagnostics GmbH, Manheim, Germany). In addition, the presence of the primary antibodies was verified in some cells by secondary polyclonal goat antibodies against mouse IgG labelled with fluorescein isothiocyanate (FITC) (Santa Cruz Biotechnology, Inc.; diluted 1:10,000). Staining in the cells was assessed using a fluorescent microscope (M205 FCA, Leica Microsystems GmbH, Wetzlar, Germany) equipped with specific wavelength filters for fluorescein-5-isothiocyanate (FITC) and 4′,6-diamidino-2-phenylindole (DAPI) and a PlanApo dry objective at 20× magnification. Images were acquired using a DFC 480 camera and processed using the Leica IM500 software. Cells processed without primary or secondary antibodies were used as negative controls. Cells expressing a signal greater than what was observed in the background negative control experiments were considered to have a positive signal. The proportion of cells containing visible molecules relative to the total number of cells was then calculated.

### IGF-I assay

IGF-I release in the culture medium was measured using an IGF-I solid phase ELISA kit with an IGF-I extract (Labor Diagnostica Nord GmbH. & Co., Nordhorn, Germany) according to the manufacturer’s instructions. Assay sensitivity was 9.75 ng/mL, and the inter- and intra-assay coefficients of variation did not exceed 14.8% and 7.3%, respectively.

### Statistical analyses

Each experiment was repeated three-times using different animal sources of granulosa cells (8–12 animals per experiment). Each experimental group was represented by four chamber-slide wells. In each well, at least 1000 randomly selected cells were assessed by a Trypan blue test and quantitative immunocytochemical assays. The amount of IGF-I in each experiment was analysed in groups of four wells per measurement. After the IGF-I assay, the values of the blank controls were subtracted from the specific values determined in the cell-conditioned medium to exclude any non-specific background values (which were less than 7% of the total values). Rates of secretion were calculated as a proportion of 1 × 10^6^ viable cells/day. Differences among groups were evaluated using one-way ANOVA followed by Tukey’s test using Sigma Plot 11.0 (Systat Software, GmbH, Erkhart, Germany). Values are presented as means ± *SEM*. Differences were considered to be statistically significant if *p* < 0.05.

## Results

**Figure 1.**
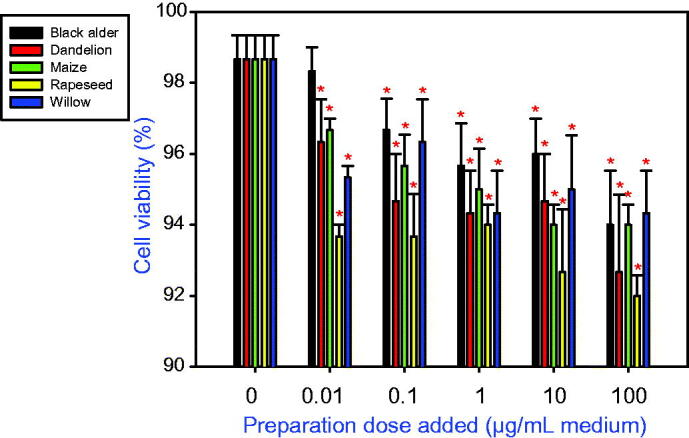
The effect of bee pollens extracted from black alder (*Alnus glutinosa*, A), dandelion *(Taraxacum officinale*, B), maize (*Zea mays*, C), rapeseed (*Brassica napus*, D), and willow *(Salix* spp., E) on the viability of cultured porcine ovarian granulosa cells. Values are presented as means ± *SEM*. Asterisks indicate significant (*p < 0.05) differences between cells treated with bee pollen and untreated cells (dose 0 ng/mL). Data from the Trypan blue test are shown.

**Figure 2.**
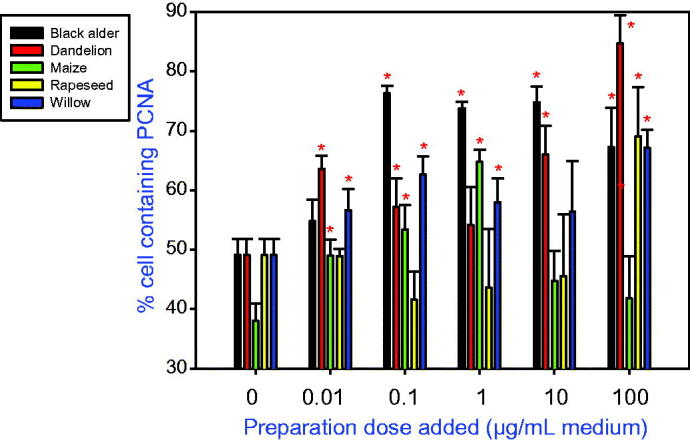
The effect of bee pollens extracted from black alder (*Alnus glutinosa*, A), dandelion *(Taraxacum officinale*, B), maize (*Zea mays*, C), rapeseed (*Brassica napus*, D), and willow *(Salix* spp., E) on the accumulation of proliferation marker PCNA in cultured porcine ovarian granulosa cells. Values are presented as means ± *SEM*. Asterisks indicate significant (*p < 0.05) differences between cells treated with bee pollen and untreated cells (0 ng/mL). Data from the quantitative immunocytochemistry analysis are shown.

**Figure 3.**
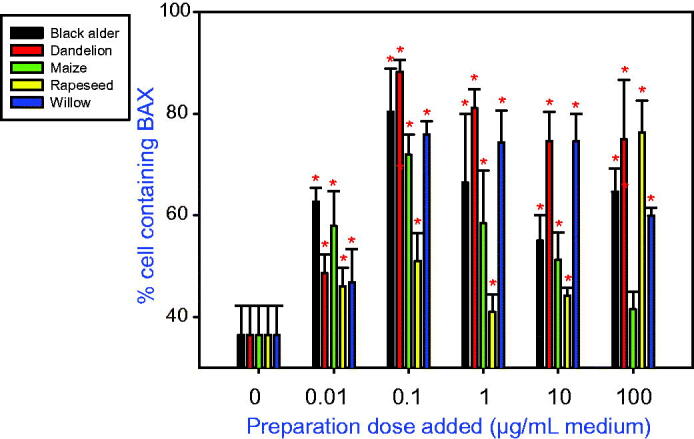
The effect of bee pollens extracted from black alder (*Alnus glutinosa*, A), dandelion (*Taraxacum officinale*, B), maize (*Zea mays*, C), rapeseed (*Brassica napus*, D), and willow *(Salix* spp., E) on the accumulation of apoptosis marker BAX in cultured porcine ovarian granulosa cells. Values are presented as means ± *SEM*. Asterisks indicate significant (*p < 0.05) differences between cells treated with bee pollen and untreated cells (0 ng/mL). Data from the quantitative immunocytochemistry analysis are shown.

**Figure 4.**
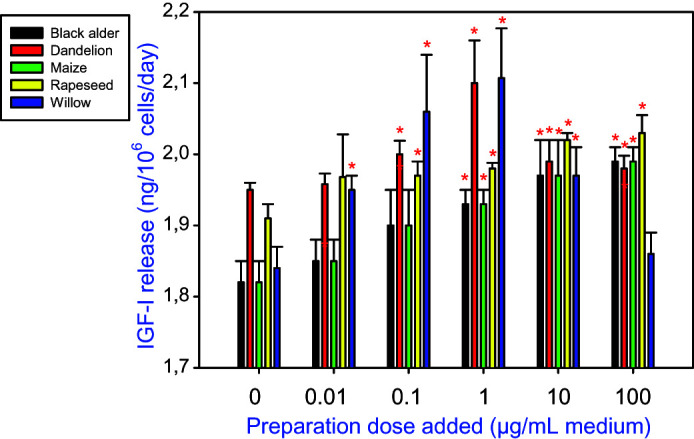
The effect of bee pollens extracted from black alder (*Alnus glutinosa*, A), dandelion (*Taraxacum officinale*, B), maize (*Zea mays*, C), rapeseed (*Brassica napus*, D), and willow *(Salix* spp., E) on IGF-I release from cultured porcine ovarian granulosa cells. Values are presented as means ± *SEM*. Asterisks indicate significant (*p < 0.05) differences between cells treated with bee pollen and untreated cells (0 ng/mL). Data from ELISAs are shown.

In cultured ovarian granulosa cells, addition of all tested types of bee pollen reduced cell viability (Figure 1) and promoted accumulation of both a proliferation marker (PCNA; Figure 2) and an apoptosis marker (BAX; Figure 3). Furthermore, the treatments were able to stimulate IGF-I release into the culture medium (Figure 4).

However, substantial species-specific differences were observed among the pollens in terms of their biological activity. When black alder pollen was added at doses of 0.1, 1, 10, or 100 µg/mL, the viability of cultured porcine granulosa cells significantly decreased (*p* < 0.05) in a dose-dependent manner. Addition of dandelion, maize, rapeseed, or willow pollen reduced cell viability at all doses. The pollens with the greatest negative effects on cell viability (i.e., those that reduced viability below 93%) were the pollens from rapeseed and dandelion, whereas the pollen of willow showed the lowest negative effects on viability. Comparing the values of viability of cells cultured with different bee pollen extracts showed that the ability of various pollens to suppress cell viability ranked as follows: rapeseed > dandelion > alder > maize > willow (Figure 1).

A significant increase in PCNA accumulation occurred after adding pollen from black alder at 0.1–100 µg/mL, dandelion at 0.01, 10, and 100 µg/mL, maize at 0.01–1 µg/mL, rapeseed at 100 µg/mL, and willow at 0.01, 0.1, and 100 µg/mL. The most potent stimulators of the expression of this proliferation marker were the pollens from dandelion, maize, and willow, which were able to promote PCNA accumulation at the lowest tested dose (0.01 µg/mL). The most effective doses were 0.1 µg/mL for black alder, 1 µg/mL for maize, and 100 µg/mL for both dandelion and willow pollens. The least potent PCNA stimulator was pollen from rapeseed which promoted PCNA accumulation only at the highest tested dose (100 µg/mL). Thus, the biological activity of bee pollens in their stimulatory action on ovarian cell proliferation ranked as follows: dandelion > willow > maize > alder > rapeseed (Figure 2).

Addition of all types of bee pollen tested also significantly (*p* < 0.05) increased accumulation of the apoptosis marker BAX within the examined cells. Addition of bee pollen originating from black alder, dandelion, maize, and willow all significantly promoted BAX accumulation at all tested doses (0.01–100 μg/mL). The most effective dose for inducing BAX accumulation among all bee pollens was 0.1 µg/mL. The least potent apoptosis stimulator was pollen from rapeseed which promoted BAX accumulation only at the highest administered dose (100 µg/mL). The promotion of cell apoptosis by pollens ranked as follows: > dandelion > alder > rapeseed > willow > maize (Figure 3).

The amount of IGF-I released into the culture medium increased after the addition of any of the tested bee pollen extracts; however, the extent of this increase was also species-dependent. Black alder pollen stimulated IGF-I release in a dose-dependent manner, which was significant at the highest doses tested (1, 10, or 100 µg/mL). Pollen originating from dandelion increased IGF-I output following a bell-shaped dose-response curve to 1, 10, and 100 µg/mL treatment, among which the most effective dose was 1 µg/mL. Maize and rapeseed pollen significantly increased IGF-I release in a dose-dependent manner at 10 and 100 µg/mL and at 0.1, 1, 10, and 100 µg/mL, respectively. The bee pollen of willow stimulated IGF-I output following a bell-shaped curve at doses of 0.1, 1, and 10 µg/mL. Willow pollen exerted the greatest influence on IGF-I accumulation as it increased IGF-I release at the lowest tested doses (0.1, 1, and 10 µg/mL) while maize expressed minimal IGF-I-promoting activity that was only significant at the highest tested doses (10 or 100 µg/mL). Thus, the biological potency of various pollens in promoting IGF-I output ranked as follows: willow > dandelion > rapeseed > maize > alder (Figure 4).

## Discussion

### Does bee pollen directly affect ovarian cell viability?

Results of our experiments demonstrated that bee pollens originating from the five tested plants substantially reduced viability of the cultured ovarian granulosa cells. This study is the first to demonstrate the adverse effects of bee pollen on ovarian cell viability. This potential effect on female reproduction should be considered before widely using bee pollen as functional food or therapeutic medicine. Other results from our experiments identified possible mechanisms of the inhibitory action of bee pollens on ovarian cell viability.

### Is the effect of pollen on cell viability due to its influence on ovarian cell proliferation, apoptosis, or both?

Our observations demonstrate the first evidence of the direct action of bee pollen on ovarian cell proliferation and apoptosis, as previous experiments using rapeseed bee pollen failed to detect direct effects (Kolesarova et al. 2011a). Increased accumulation of apoptosis-related proteins BAX and caspase-3 in the ovaries of rats fed rapeseed bee pollen had previously been reported (Kolesarova et al. 2013); however, these experiments failed to show whether bee pollen affects ovarian cell apoptosis directly or instead exerted apoptotic effects by impacting upstream regulatory pathway components. Nevertheless, these studies indicated that bee pollen could have pro-apoptotic properties. In our experiments, all tested bee pollens were able to promote both proliferation (PCNA accumulation) and apoptosis (accumulation of BAX). These increases in cell proliferation and death suggest that bee pollen can promote ovarian cell turnover. Similar stimulatory actions on cultured ovarian cell turnover has been previously reported: promotion of both proliferation and apoptosis was observed in known regulators of ovarian functions such as follicle stimulating hormone (Sirotkin et al. 2014, 2017), leptin (Sirotkin et al. 2012), IGF-I and ghrelin (Sirotkin et al. 2014), some siRNAs controlling ovarian cell protein kinases (Sirotkin et al. 2010), iron (Kolesarova et al. 2011b), and the plant isoflavone daidzein (Sirotkin et al. 2017) which is present in bee pollen (Denisow and Denisow-Pietrzyk 2016; Cornara et al. 2017; Kieliszek et al. 2018). Therefore, daidzein is one component that could be responsible for the effects of bee pollen on the ovary. The effects of bee pollen and the effects of listed hormonal regulators of ovarian cell turnover are similar, indicating that bee pollen and its constituents could be synergists or even replacements for classical hormonal stimulators of female reproduction. However, this hypothesis requires stronger validation through further *in vitro* and *in vivo* studies.

The ratio of cell proliferation to apoptosis determines cell turnover rate, ovarian follicle population viability, ovarian follicle fate (growth and development or atresia), and ultimately, female fecundity. Although bee pollen promoted both cell proliferation and apoptosis in our experiments, overall, bee pollen treatment decreased in cell viability, i.e., increased in cell death. This suggests that bee pollen-induced changes in the proliferation-to-apoptosis ratio, resulting in the dominance of apoptosis over proliferation. Increased apoptosis (programmed cell death) could, therefore, cause reduced viability in cells treated with bee pollens as observed in our experiments.

### Is the influence of bee pollen on viability, proliferation, and apoptosis due to its impact on IGF-I release?

In our experiments, treatment with the tested bee pollens stimulated the release of IGF-I into the medium by cultured ovarian cells. This is consistent with previous reports on the action of rapeseed pollen on IGF-I levels in rat blood (Kolesarova et al. 2013) and IGF-I release by cultured porcine ovarian cells (Kolesarova et al. 2011a). However, in the report of Kolesarova et al. (2011a), bee pollen actually inhibited IGF-I output. The ability of IGF-I to mediate the effects of external factors on the ovary, promote ovarian cell proliferation, inhibit ovarian cell apoptosis, promote cell and ovarian follicle viability, and promote release of other ovarian hormones is well documented (Quirk et al. 2004; Sirotkin 2011, 2014; Shimizu 2016). Therefore, it is possible that the ability of bee pollen to promote ovarian cell proliferation could be mediated inducing release of IGF-I, which is a stimulator of cell proliferation. However, an increase in IGF-I release cannot explain the increase in apoptosis and decrease in cell viability observed in our study. Similarly, we observed bee pollen-induced decreases in the release of IGF-I, a known promoter of ovarian steroidogenesis; this cannot explain the unaltered or even increased steroidogenesis reported by Kolesarova et al. (2011a, 2013). Therefore, the bee pollen’s effects on the ovary are mediated in part by the changes it causes in IGF-I release.

### Does the plant species from which bee pollen originates impact its effects on ovarian cells?

Our study is the first comparison of the effects of bee pollen originating from different plant species on ovarian functions. This is the first demonstration that reproductive effects of bee pollen are dependent on the plant species from which the pollen originated. Although all tested bee pollen types had similar overall influences on ovarian cell viability, proliferation, apoptosis, and IGF-I release, substantial interspecies differences were found on the magnitudes of their biological activity as well as on the dose-dependency of these effects.

The most potent inhibitors of cell viability were bee pollens from dandelion and rapeseed, whereas pollen from willow had the lowest activity. The most potent stimulators of cell proliferation were pollens from dandelion and maize, while the least potent stimulator was rapeseed pollen. Apoptosis was strongly promoted by pollens originating from black alder, dandelion, maize, and willow, whereas the least potent apoptosis stimulator was pollen from rapeseed. The low biological activity of rapeseed bee pollens could explain the lack of impact of this pollen on porcine ovarian cell proliferation and apoptosis observed in previous experiments (Kolesarova et al. 2011a).

The most potent IGF-I stimulator was willow pollen, while the least potent was maize pollen. Therefore, no tested pollen had the strongest effect on all the functional endpoints measured. For example, while rapeseed had the most potent effects on cell viability, it was the least potent regulator of cell proliferation and apoptosis. On the other hand, willow pollen was the most potent IGF-I stimulator but it had the least potent effects on cell viability. The dandelion pollen showed the strongest action on most (3 out of 4) of the measured parameters.

In addition to biological activity, the dose-dependent effects of bee pollen on the measured parameters also depended on pollen origin. Only cell viability decreased in a dose-dependent manner after the addition of each pollen; however, substantial species-specific differences was observed in the dose-response curves to bee pollen for the other three endpoints. The effects of some bee pollens on cell proliferation, apoptosis, and IGF-I release were proportional to the pollen dose added, while other pollens affected these parameters in a bell-shaped dose-response manner. Such bell-shaped dose-response curves indicate the existence of negative feedback mechanisms preventing hypertrophied responses to selected bee pollens. In other pollens which continued to affect cells in a dose-dependent manner even up to the highest concentrations tested, no evidence for the existence of such negative feedback mechanisms was found. Species-specific differences among the different pollens in terms of both biological activity and dose-response curves of their effects on different parameters indicate that bee pollens have relatively independent effects on different ovarian functions. This may indicate that bee pollens originating from different plants have different constituents and/or different mechanisms of action on different ovarian functions.

Species-specific differences in the biological activities of the bee pollens analysed in our study should be considered during the potential application of bee pollens in apitherapy or in the preparation of functional foods containing bee pollen. In our experiments, pollens originating from dandelion and maize seemed to be more preferable for such uses, whereas rapeseed pollens had lower overall biological activities, making them less preferable. Therefore, our observations indicate that the pollen species to be used in various applications should be selected based on its particular target process.

## Conclusions

Taken together, our observations are the first to demonstrate that bee pollen can directly reduce ovarian cell viability and upregulate ovarian cell proliferation, apoptosis, and IGF-I release. Bee pollen-induced reductions in cell viability may be due to increased ovarian cell apoptosis, whereas pollen-induced increases in cell proliferation might be the result of increased IGF-I release. Furthermore, although all the tested bee pollens had similar overall effects on the measured ovarian cell parameters, our observations are the first to show that the biological potency and dose-response curves of bee pollens depend on their plant species of origin. The effects of bee pollen on ovarian cell functions and the species-specific differences in these effects should be considered during the application of bee pollen in nutrition and medicine.
